# Specialized dermatological-rheumatological patient management improves diagnostic outcome and patient journey in psoriasis and psoriatic arthritis: a four-year analysis

**DOI:** 10.1186/s41927-021-00217-z

**Published:** 2021-10-30

**Authors:** Jana Ziob, Charlotte Behning, Peter Brossart, Thomas Bieber, Dagmar Wilsmann-Theis, Valentin Sebastian Schäfer

**Affiliations:** 1 Department of Dermatology and Allergy, University Hospital of Bonn, Venusberg-Campus 1, 53127 Bonn, Germany; 2 Department of Medical Biometry, Informatics and Epidemiology, University Hospital of Bonn, Bonn, Germany; 3 Clinic of Internal Medicine III, Department of Oncology, Hematology, Rheumatology and Clinical Immunology, University Hospital of Bonn, Bonn, Germany

**Keywords:** Dermatological-rheumatological patient management, Psoriasis, Psoriatic arthritis, Patient journey, Diagnostic outcome

## Abstract

**Background:**

Management of psoriasis patients with arthralgia suffering from suspected psoriatic arthritis (PsA) requires an interdisciplinary approach involving dermatologists and rheumatologists. The aim of the study was to analyze the specialized dermatological-rheumatological management of these patients before and after foundation of a PsA center.

**Methods:**

A retrospective cohort study of all dermatological-rheumatological consultations during two periods was conducted. Period one, from April 1st, 2016 to February 28th, 2018 versus period two, from March 1st, 2018 to January 31st, 2020, after foundation of a PsA center. Clinical data on patient characteristics including psoriasis subtypes, clinical symptoms and signs, disease activity scores, classification criteria and comorbidities as well as patient journey were extracted and analyzed.

**Results:**

Four hundred four consultations were studied. Close collaboration in a PsA center lead to a relevantly shortened patient journey concerning rheumatological complaints: period 1: median (IQR): 36.0 (10.0–126.0) months, period 2: median (IQR): 24.0 (6.0–60.0) months. Established scores and classification criteria such as GEPARD or CASPAR did not assist in diagnosis of PsA. Arthralgia (*p* = 0.0407), swollen joints (*p* = 0.0151), morning stiffness (*p* = 0.0451) and dactylitis (*p* = 0.0086) helped to distinguish between osteoarthritis and PsA.

**Conclusions:**

Clinical signs and symptoms, scores and classification criteria usually assessed were less helpful than expected in diagnosis of PsA. Close collaboration in a specialized PsA center yielded the fastest way of diagnosis.

## Background

Psoriasis is a common chronic inflammatory immune-mediated skin disorder which affects about 2–3% of adults in Western populations and therefore has a substantial socio-economic significance [1]. Psoriatic Arthritis (PsA), which occurs in up to 40% of patients affects peripheral joints, the spine, and tendons [2–4]. It is estimated that 29% of psoriasis patients seen by dermatologists suffer from undiagnosed PsA [5]. This can be due to dermatologists primarily concentrating on skin lesions, rather than joint complaints [3]. In most cases, psoriasis develops 10–12 years before the occurrence of PsA, underlining the important role of dermatologists in recognizing joint disorders for timely referral and adequate therapy [3, 6, 7]. Alternatively, in 14–20% of PsA patients, arthritis can occur before or simultaneously with psoriasis [8]. Other forms of arthritis such as osteoarthritis (OA) or rheumatoid arthritis sometimes imitate PsA, therefore correct diagnosis is of high importance.

Despite the assumed high importance of interdisciplinary collaboration in dermatology-rheumatology, the clear evidence for it has not been explored significantly yet. Therefore, we designed a study at our University Hospital of Bonn to compare two specific periods, one before and one after the foundation of a specialized PsA center, enabling close dermatology-rheumatology cooperation. This is the first study focusing on the role of early dermatological-rheumatoloicaly management of patients with psoriasis and suspected PsA before and after establishment of a specialized center considering the patient journey as well as the diagnostic outcome.

## Methods

### Study design and participants

We performed a retrospective cohort study of the interdisciplinary dermatological-rheumatological cooperation and management of patients with psoriasis, PsA, other rheumatic diseases and non-rheumatic diseases by comparing two specific periods: period 1, from April 1st, 2016 to February 28th, 2018 versus period 2, from March 1st, 2018 to January 31st, 2020, after foundation of a specialized PsA center. Once a week a consultation shared by dermatologist and rheumatologist took place in the department of rheumatology, with examination of dermatological patients suspected of suffering from rheumatological diseases.

### Data collection

Detailed clinical data was obtained from Dedalus ORBIS™, which is used as main electronic health record system at the University Hospital of Bonn. We analyzed all consultation records between both departments. The study followed the principles of the Declaration of Helsinki and the guidelines of Good Clinical Practice and was approved by the local ethics committee of the University Hospital of Bonn (Ethik-Kommission der Medizinischen Fakultät Bonn – Lfd. Nr. 100/20). Due to the retrospective design of the study, the need for informed consent was waived by the ethics committee of the University Hospital of Bonn, Germany (Chairman Prof. Dr. Racké).

General characteristics collected during the study included patient’s age, sex, body mass index, comorbidities, history of smoking and laboratory parameters such as rheumatoid factor (RF), anti-cyclic citrullinated peptide antibody (anti-CCP), C-reactive protein (CRP) and more. Furthermore, we assessed subtype of psoriasis, family history, nail, scalp and rima ani involvement. We also included the following established disease scores: PASI (Psoriasis Area and Severity Index), DLQI (Dermatology Life Quality Index), GEPARD (German Psoriatic Arthritis Diagnostic Questionnaire) and CASPAR (Classification Criteria for Psoriatic Arthritis). Other collected data included duration of skin lesions and rheumatological complaints, suspected diagnosis and duration from consult request until consultation. We also assessed all pre-diagnosed and first diagnosed diseases, clinical signs and symptoms and fulfillment of classification criteria. CASPAR classification criteria were applied for PsA.

### Statistical analysis

Exploratory data analysis was performed with “R”, version 4.0.2 [9]. Categorical variables were expressed as absolute and relative frequencies, continuous variables in mean ± standard deviation (SD) and median + interquartile range (IQR). Categorical variables were examined using Chi-Square test or Fisher’s exact test, when absolute frequencies were below five. Continuous variables were compared by using Wilcoxon tests when normality could not be assumed. Descriptive statistics are presented, as *p* < 0.05 was considered significant. For some parameters subgroup analyses were performed based on the presence of psoriasis, PsA or OA.

## Results

Over the four-year period, 404 dermatological consultations were answered by the department of rheumatology. 61 out of 587 rheumatological consultations were addressed in the first period before the establishment of a PsA center and 343 out of 1104 rheumatological consultations in the second period. Excluding follow-up consultations, 373 patients including 165 men and 208 women with a mean age of 52.3 (±15.4) years were studied. Coexisting diseases were counted separately. Other rheumatological and non-rheumatological diseases except osteoarthritis were not analyzed.

### Patient characteristics

Analysis of our whole cohort confirmed the presence of psoriasis in 218 of 373 patients, while PsA was diagnosed in 103 cases (47.25%). Thirty-nine patients with PsA (37.86%) had a positive family history of psoriasis. Body mass index (BMI) and gender distribution did not differ between these two groups. Patients with psoriasis displayed a higher prevalence, compared to PsA (46.79% versus 39.81%). In both groups, elevated RF-Levels (Psoriasis: 5.96%, PsA: 5.83%) and anti-CCP antibodies (Psoriasis: 2.75%, PsA: 2.91%) were found in a comparable distribution (Table 1).

**Table 1:** Patient characteristics in patients with psoriasis and psoriatic arthritis over both periods

Parameter	Psoriasis	Psoriatic arthritis
Number, n in total	218	103
Age (years) (mean ± SD)	49.9 (±14.70)	52.0 (±13.90)
Gender: men, n (%)	115 (52.75)	56 (54.37)
Gender: women, n (%)	103 (47.25)	47 (45.63)
BMI (kg/m^2^) (mean ± SD)	29.1 (±6.50)	29.4 (±6.40)
Elevated CRP-Level (≥ 5 mg/l), n (%)	44 (20.18)	20 (19.42)
Elevated anti-CCP antibodies (≥ 8,0 U/ml), n (%)	6 (2.75)	3 (2.91)
Elevated RF-Level (≥ 14 IU/ml), n (%)	13 (5.96)	6 (5.83)
Smoking, ever, n (%)	102 (46.79)	41 (39.81)
Smoking, never, n (%)	60 (27.52)	31 (30.10)
Allergies, n (%)	80 (36.70)	32 (31.07)
Initial diagnosis, n	–	43

Abbreviations: *n* number, *BMI* body mass index, *CRP* C-reactive protein, *anti-CCP antibody* anti-cyclic citrullinated peptide antibody, *RF* rheumatoid factor

### Disease activity scores and classification criteria

Comparison of both the periods revealed diagnosis of PsA in 103 patients with psoriasis, while the initial diagnosis confirmed it in only 43 patients. Clinical signs and symptoms were assessed anamnestically. In the PsA group, all patients reported of arthralgia, while 56 patients (54%) reported of previously swollen joints and 26 (24%) of tender joints. 29 patients (28%) reported morning stiffness lasting over 60 min. In Psoriasis group, 210 patients (96%) suffered from arthralgia, 92 (42%) reported of previously swollen joints and 48 (22%) from previously tender joints. Suspected PsA was confirmed in 78 cases out of 277 patients (28.16%). PsA was diagnosed in 25 individuals who had no prior suspicion of the disease (24.27%). Results from PASI, DLQI and GEPARD as well as CASPAR classification criteria were similar in both groups (Table 2).

**Table 2:** Disease activity scores and classification criteria in patients with psoriasis and psoriatic arthritis at the time of consultation

Element / Disease	Psoriasis patients	Psoriatic arthritis patients
Number, n in total	218	103
PASI (mean ± SD)	12.1 (±8.4)	11.8 (±9.6)
DLQI (mean ± SD)	14.5 (±6.8)	15.9 (±6.8)
GEPARD (mean ± SD)	6.7 (±3.3)	8.2 (±2.7)
CASPAR (mean ± SD)	5.1 (±1.8)	5.8 (±1.7)

Abbreviations: *n* number, *PASI* Psoriasis Area and Severity Index, *DLQI* Dermatology Life Quality Index, *GEPARD* German Psoriatic Arthritis Diagnostic Questionnaire, *CASPAR* Classification Criteria for Psoriatic Arthritis

### Psoriasis subtypes

The most prevalent type of psoriasis in patients with PsA was plaque psoriasis reported in up to 86.4% of patients. Concomitant nail, scalp and rima ani involvement was observed with similar frequencies in psoriasis and PsA groups (Table 3). Of all measurements of PsA patients (103), 69 patients (66.99%) reported suffering from skin manifestations before joint complaints appeared, 7 patients (6.80%) stated joint complaints before developing psoriatic skin lesions and 8 patients (7.77%) developed skin and joint complaints simultaneously. Nineteen patients were not assessed.

**Table 3:** Psoriasis subtypes in patients with psoriatic arthritis

Psoriasis forms	n (%)
in total	103 (100)
Isolated plaque psoriasis	89 (86.41)
Isolated pustular psoriasis	8 (7.77)
Isolated inverse psoriasis	1 (0.97)
Isolated scalp psoriasis	4 (3.88)
Isolated nail psoriasis	1 (0.97)
Concomitant involvement of the scalp	51 (49.51)
Concomitant involvement of the rima ani	50 (48.54)
Concomitant involvement of the nails	44 (42.72)

Abbreviations: *n* number

### Comorbidities

The prevalence of comorbidities was high in both groups, especially, concerning cardiovascular, gastrointestinal and neurological/psychiatric diseases. Cardiovascular diseases were the most commonly observed comorbidity in psoriasis patients (37.50%) while ophthalmological diseases were the least common observed comorbidity (2.50%). In PsA patients neurological and psychiatric diseases were most commonly observed (34.95%) whereas ophthalmological diseases were equally uncommon (2.91%). Moreover, we noticed significant differences in neurological and psychiatric diseases between the psoriasis and PsA group (*p* = 0.002843) (Table 4).

**Table 4:** Comorbidities in patients with psoriasis and psoriatic arthritis

Parameter	Psoriasis, n (%)	Psoriatic arthritis, n (%)
Number, n in total	120 (100)	103 (100)
Viral infectious diseases	9 (7.50)	6 (5.83)
Previously diagnosed malignancy	16 (13.33)	16 (15.53)
Cardiovascular diseases	45 (37.50)	31 (30.10)
Pulmonary diseases	13 (10.83)	11 (10.68)
Diabetes mellitus	15 (12.50)	13 (12.62)
Peripheral vascular diseases (venous and arterial)	12 (10.00)	5 (4.85)
Gastrointestinal diseases	31 (25.83)	28 (27.18)
Ophthalmological diseases	3 (2.50)	3 (2.91)
Neurological and psychiatric diseases	20 (16.67)	36 (34.95)

Abbreviations: *n* number

### Symptoms and signs of psoriatic arthritis and osteoarthritis

In 55 patients suffering from suspected PsA, OA was diagnosed. Fifty of these were first diagnosed, six in period 1 and 44 in period 2. All patients with PsA (100%) and nearly all with OA (95%) reported of arthralgia, while swollen joints were observed in 54% patients in PsA and 33% patients in OA. Tender joints were presented in 25% cases in PsA and 11% cases in OA. In OA patients, no enthesitis or dactylitis was observed in contrast to PsA. Significant differences were detected in arthralgia (*p* = 0.0407), swollen joints (*p* = 0.0151), morning stiffness of the digitis (*p* = 0.0451) and dactylitis (*p* = 0.0086) (Table 5).

**Table 5:** Reported symptoms and signs of patients with psoriatic arthritis and osteoarthritis

Element	Psoriatic arthritis, n (%)	Osteoarthritis,n (%)	*P*-value*
Reported symptoms			
Number, n in total	103 (100)	55 (100)	
Arthralgia	103 (100)	52 (94.55)	0.0407 ‡
Morning stiffness of digits	29 (28.16)	7 (12.73)	0.0451 †
Swollen joints	56 (54.37)	18 (32.73)	0.0151 †
Tender joints	26 (25.24)	6 (10.91)	0.0539 †
Pain insomnia	13 (12.62)	6 (10.91)	0.9533 †
Alleviation with movement	4 (3.88)	2 (3.64)	1.0000 ‡
Clinical signs			
Number, n in total	26 (25.24)	1 (1.82)	< 0.0001‡
Joint deformities	3 (2.91)	1 (1.82)	1.0000 ‡
Positive Gaenslen’s Squeeze Test of MCP joints	8 (7.77)	0 (0)	0.0512 ‡
Enthesitis	3 (2.91)	0 (0)	0.5520 ‡
Dactylitis	12 (11.65)	0 (0)	0.0086 ‡

* *P*-values are given for Chi-Square Test (†) and Fishers-Exact-Test (‡). Abbreviations: *n* number, *MCP* metacarpophalangeal

### Patient journey before and after the foundation of a psoriatic arthritis center

A number of 372 consultations were assessed over both periods, 52 in period 1 (14%) and 320 in period 2 (86%) – before and after the foundation of PsA center. Despite the fact that 6-fold increase in consultation was reported in period 2, the distribution of consultation type (day patient versus inpatient versus outpatient) was altered. The outpatient clinic played the greatest role in period 2 (139 consultations, 43%) while it played the subordinated role in period 1 (7 consultations, 13%).

**Fig. 1:**
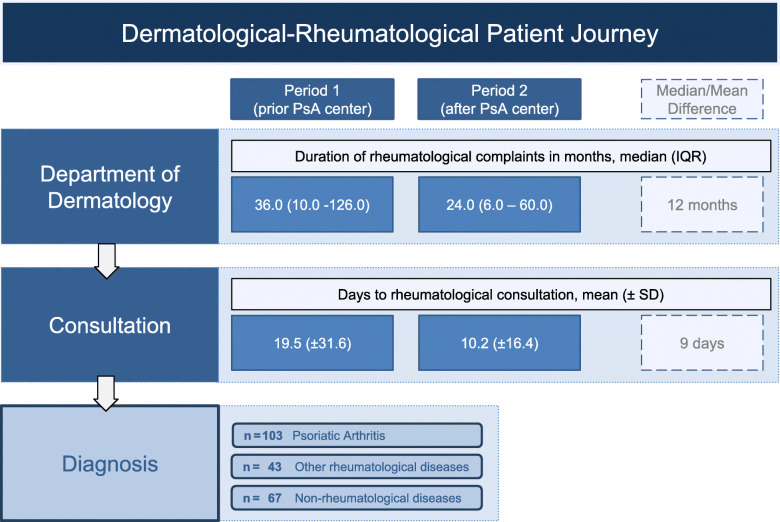
Dermatological-Rheumatological patient journey over both periods

During this period, 103 patients were diagnosed with PsA in total, out of which 15 were consulted in period 1, while 88 in period 2. These patients suffered from rheumatological complaints for 36.0 (IQR:10.0–126.0) median months in period 1 and 24.0 (IQR:6.0–60.0) median months in period 2. No significant difference was observed (*p* = 0.1527) using the Wilcoxon test but a trend for shortened duration of about 12 months was noted making it clinically relevant (Fig. 1).

Period 1: prior foundation of psoriatic arthritis center, period 2: after foundation of psoriatic arthritis center, duration of rheumatological complaints in months until presentation at the department of dermatology, days to rheumatological consultation, assessed median/mean differences, respective diagnoses made.

Overall, 209 patients out of all assessed patients (373) complained about skin manifestations before the begin of arthralgia, 43 patients reported arthralgia before skin manifestations and 30 developed skin lesions simultaneously to joint complaints. Data on analysis of both periods showed the need of less median months (180 (IQR:84–360)) in period 2 compared to period 1 (204 (IQR:60–336)) to report the beginning of skin lesions until joint complaints occurred. Developing joint complaints before skin manifestation took 84 (IQR:44–96) median months in period 1 and 21 (IQR:5.8–28.5) median months in period 2.

In PsA, 69 patients complained about skin lesions before the begin of arthralgia, seven patients reported of arthralgia before the onset of skin lesions and 8 developed psoriatic skin lesions simultaneously to joint complaints. Focusing on both analyzed studied periods, in period 1 it took 288 (IQR: 153.0–438.0) median months and in period 2 264 (IQR: 120.0–360.0) median months from the onset of skin lesions until joint complaints occurred. Developing joint complaints before onset of skin lesions took 48.0 (IQR: 25.0–85.0) median months in period 2.

During both periods, in 78 out of 277 cases, suspected PsA was confirmed. In period 1, PsA was diagnosed in 10 out of 32 patients (31.25%) with suspected PsA, and in period 2, 68 out of 245 patients (27.8%) with suspected PsA were diagnosed with PsA. In 25 out of 103 cases (24.27%) over both periods in total, PsA was diagnosed without prior suspicion.

In period 1, one of two patients who was first diagnosed with PsA fulfilled the CASPAR classification criteria whereas in period 2 all 41 PsA patients fulfilled respective classification criteria. Using the Chi-Square Test for meeting PsA classification criteria (*p* = 0.03458) over both periods, significant differences were observed.

## Discussion

To date, very few studies have evaluated the feasibility of setting up a specialized PsA center. In fact, this kind of structure with very close cooperation of dermatologists and rheumatologists is limited mostly to university hospitals. The aim of our PsA center is to optimize the medical care for psoriasis patients with suspected PsA in a team-oriented approach.

The direct referral of patients with suspected PsA to the specialized PsA center can help to overcome the confusion between arthritis diagnosis and treatment in patients, thus increasing the attention of patients to management and follow-up in this specialized center. The PsA center’s most critical priorities are the early diagnosis and treatment of PsA. PsA, if left untreated, can result in irreversible joint deformity causing immobility and worsening the overall well-being issues [10].

Differential diagnosis is sometimes complex and early management is difficult, requiring the aid of a rheumatologist. There are many studies underlying the delay in diagnosis and treatment of PsA, which typically leads to different orthopedic operations until these patients finally are diagnosed [10, 11]. Previous studies also suggested, that there are significant deviations in clinical management and follow-up of patients with psoriasis and PsA, depending on the specialist they initially choose to see, often leading to misdiagnosis and sometimes loss of diagnosis, decreasing the compliance of the patient [12–14]. In addition, in a specialized PsA center, rheumatologists are acquainted with topical therapies used in dermatology as well as with the dermatological view of systemic therapies concerning the efficacy in plaque psoriasis. In the PsA center, severity of skin changes are carefully assessed by the dermatologist and reported to the rheumatologist, so that both can decide on a common therapeutic algorithm, tailored for each patient. Furthermore, we can learn more about paradoxical reactions on the skin due to different biological therapies, which could be addressed in an interdisciplinary setting.

In patients with PsA, a positive family history for psoriasis was present in 37.86%. Although, patients with PsA are usually negative for RF, elevated levels were found in 6 cases (5.83%) [15]. A similar percentage was observed in patients with psoriasis probably relating to similar presence in the general population [16].

Our data did not show significant differences in PASI, DLQI, GEPARD and CASPAR scores between patients with psoriasis and PsA. The GEPARD questionnaire (mean ± SD: Psoriasis = 6.7 (±3.3), PsA = 8.2 (±2.7)) and the CASPAR classification criteria (mean ± SD: Psoriasis = 5.1 (±1.8), PsA = 5.8 (±1.7)) which are commonly used, were not found to be helpful for differentiating between psoriasis and PsA. This is commonly observed in clinical practice, although the literature suggests a better diagnostic yield [17, 18]. Despite this, the higher level of DLQI in patients with PsA, which primarily affects the impact of the skin symptoms on health-related quality of life, indicated a greater degree of impairment in those patients [19]. On the other hand, the dermatologist should not stop asking for joint complaints in psoriasis patients as PsA is often developed in the later history of psoriasis [4].

Plaques psoriasis (86.41%) was the most common type of psoriasis in PsA patients. Current literature underlines our observation, as a 2–3-fold higher risk of developing PsA in patients with scalp lesions, nail dystrophy and perianal lesions is described [20]. Our patients with PsA reported scalp involvement in 49.51%, rima ani involvement in 48.54% and nail involvement in 42.72%, so no specific form of psoriasis was found to be linked to PsA.

Our study yielded a similar increase in prevalence of comorbidities as published, highlighting cardiovascular diseases, gastrointestinal, psychiatric and neurological diseases in psoriasis and PsA [21].

A total of 43 patients out of 373 (11.5%) were diagnosed with another rheumatological disease than PsA, underlining the significance of the dermatological-rheumatological approach not only in PsA.

Non-rheumatological diseases were diagnosed in 67 patients out of 373 (18%). In 55 (15%) patients OA was diagnosed, which was first diagnosed in 50 (13%) patients whereas PsA was diagnosed in 103 cases (28%). This underlines the complexity of differentiating OA from PsA for dermatologist. Although, we assessed significant differences in the history of arthralgia (*p* = 0.0407), swollen joints (*p* = 0.0151), morning stiffness of the digits (*p* = 0.0451) and dactylitis (*p* = 0.0086), tender joints and pain insomnia did not play prominent roles. These symptoms and clinical signs can help to distinguish between OA and PsA but further diagnostics is required. This emphasizes the difficulty for dermatologists in interpreting joint complaints and that is not described in the literature. Future trials will need to focus more on diagnostic tools such as ultrasound, facilitating the diagnosis of arthritis. An additional validated questionnaire could be another future vision improving the diagnostic yield, but is obviously quite complex, as shown by our results concerning CASPAR classifications criteria and the GEPARD questionnaire.

Data on patient journey in both the periods show that the time taken for diagnosis by the rheumatologist was about 12 months shorter in period 2 after the foundation of the PsA center compared to period 1, which is clinically significant, in order to prevent irreversible damage of the joint.

There are several examples of collaboration between different departments such as uveitis clinics or reproduction and pregnancy clinics in which the patient is seen by various experts for chronic medical complaints [19, 22, 23]. These experiences demonstrate a significant diagnostic and therapeutic benefit. Positive aspects of these specialized centers include avoiding excessive laboratory testing, minimizing the number of visits, efficient decision-making and efficient flow of knowledge leading to fastened diagnosis and procedures for patient management.

Dermatologists and rheumatologists mutually are involved in the management of all overlapping disorders, notably PsA, as they specifically focus on their area of expertise. They see the patients in their respective offices at different times. This form of practice model resulted in the lack of communication and teamwork between specialists [24]. Our practice model not only decreased the duration of rheumatological complaints until request in period 2 but also significantly reduced the days required from consultation request until the consultation. In period 2, patients were seen in average 9 days earlier by the rheumatologist. Although, not investigated in this trial, it is imaginable, that the interdisciplinary consultation is not as time consuming as the single visits of each physician.

The limitations of the study are in regards to the retrospective and the monocentric study design. Future prospective studies are necessary in order to confirm our observations.

One of the greatest benefits of the PsA center is also the inspiration for research provided by the collaboration of both dermatologists and rheumatologists.

It is, to the best of our knowledge, the first study comparing patient management before and after foundation of a joint, dual center focusing on patients with psoriasis and PsA. Our conclusions complement the findings of other research groups worldwide. The importance of service delivery in interdisciplinary teams is illustrated by our data in the taking control of complicated or complex patients, suffering from more than one disease into account.

## Conclusions

The results of our study confirm the importance of a multidisciplinary university dermatological-rheumatologicaly management in a specialized PsA center. Time to rheumatological examination and time to diagnosis was significantly decreased. These data form the concept of a model that can enhance medical treatment through close interdisciplinary cooperation, which should be implemented nationally and globally. Further research showing the value of such a centered care in greater patient numbers is obligatory.

## Data Availability

The data used and/or analyzed during the current study are included in this published article. Additional data are available from the corresponding author on reasonable request.

## Abbreviations

Anti-CCP antibody: Anti-cyclic citrullinated peptide antibody
BMI: Body mass index
CASPAR: Classification Criteria for Psoriatic Arthritis
CRP: C-reactive protein
DLQI: Dermatology Life Quality Index
GEPARD: German Psoriatic Arthritis Diagnostic Questionnaire
IQR: Interquartile range
MCP: Metacarpophalangeal
N: Number
OA: Osteoarthritis
PASI: Psoriasis Area and Severity Index
PsA: Psoriatic arthritis
RF: Rheumatoid factor
SD: Standard deviation
